# Recent insights into the therapeutic strategies targeting the pseudokinase PTK7 in cancer

**DOI:** 10.1038/s41388-024-03060-x

**Published:** 2024-05-21

**Authors:** Charlotte Dessaux, Laetitia Ganier, Louis Guiraud, Jean-Paul Borg

**Affiliations:** 1 Aix Marseille Univ, CNRS, INSERM, Institut Paoli-Calmettes, CRCM, Equipe labellisée Ligue ‘Cell polarity, Cell signaling and Cancer’, Marseille, France; 2 Institut Universitaire de France, Paris, France; 3 Present Address: adMare BioInnovations, Vancouver, BC Canada

**Keywords:** Oncogenes, Extracellular signalling molecules

## Abstract

The generation of drugs counteracting deregulated protein kinases has been a major focus in cancer therapy development. Breakthroughs in this effort have produced many therapeutic agents to the benefit of patients, mostly through the development of chemical or antibody-based drugs targeting active kinases. These strategies are challenged when considering catalytically inactive protein kinases (or pseudokinases), which represent 10% of the human kinome with many of relevance in cancer. Among the so-called pseudotyrosine kinases, the PTK7 receptor tyrosine kinase (RTK) stands as a bona fide target overexpressed in several solid tumors and hematological malignancies and linked to metastasis, poor prognosis, and resistance to treatment. Despite the lack of catalytic activity, PTK7 has signaling capacities through heterodimerization with active RTKs and offers pharmacological targeting opportunities through its inactive kinase domain. Moreover, PTK7-targeting strategies based on antibody-drug conjugates, aptamers, and CAR-T cell-based therapies have demonstrated encouraging results in preclinical and clinical settings. We review the most recent data assigning to PTK7 a prominent role in cancer progression as well as current preclinical and clinical targeting strategies against RTK family pseudokinases including PTK7.

## Introduction

**Fig. 1:**
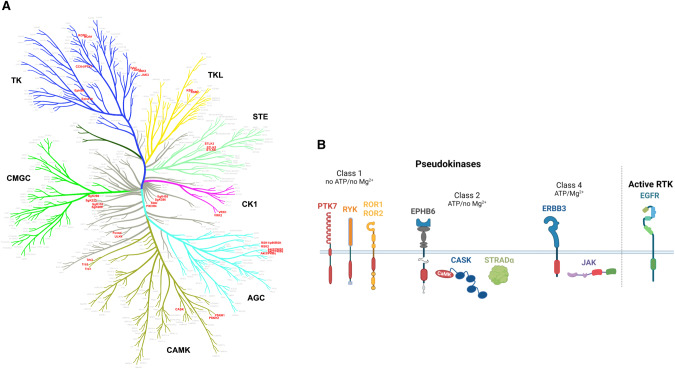
Pseudokinases in the human kinome. **A** Dendogram of human protein kinases generated with CORAL (Clustering, Orthology, and Recognition Analysis). Pseudokinase domains are colored in red. **B** Classification of pseudokinases based on their ability to bind or not ATP and Mg^2+^. The pseudokinase domains are depicted in red, while the active kinase domains are colored in green. The figure was created with BioRender.com.

Protein kinases play pivotal roles in nearly every signaling pathway. Fundamental to their cellular functions are their reliance on reversible phosphorylation events in response to extracellular stimuli, a key process revealed by Edmond Fisher and Edwin Krebs who were awarded the Nobel Prize in Medicine in 1992 for their ground-breaking discovery. Protein kinases are endowed with the capacity to transfer phosphate groups to specific residues present in proteins, lipid, carbohydrates or nucleotides, and to interact with a variety of partners (ligands, substrates, scaffold proteins) required for their signaling functions. Within the ensemble of human protein kinases (referred as the human kinome) comprising 538 proteins in total, about 11% (58 kinases) are atypical members called dead kinases or pseudokinases as initially named by Manning et al., which represent a subgroup lacking catalytic activity and phospho-transfer to substrates, and of growing interest in cancer biology and treatment (Fig. 1) [1].

For a long time, pseudokinases were not considered as priority targets in cancer drug development for the two main reasons that their oncogenic role remained poorly appreciated and that their weak or absent enzymatic activity made the development of inhibitors challenging. Nevertheless, research on membrane-bound ERBB3 and cytoplasmic JAK2 pseudokinases have opened novel perspectives as those were found mutated and acting as major oncogenes in malignant hemopathies and solid tumors, respectively. Moreover, potent chemical inhibitors or antibodies able to counteract their deleterious signaling functions have since demonstrated benefits to patients [2–4]. While the mode of action of pseudokinases remains poorly understood in general, their disease association and druggability are now largely recognized, prompting interest on their functions and on benefit in their targeting (as recently reviewed in [3]).

**Fig. 2:**
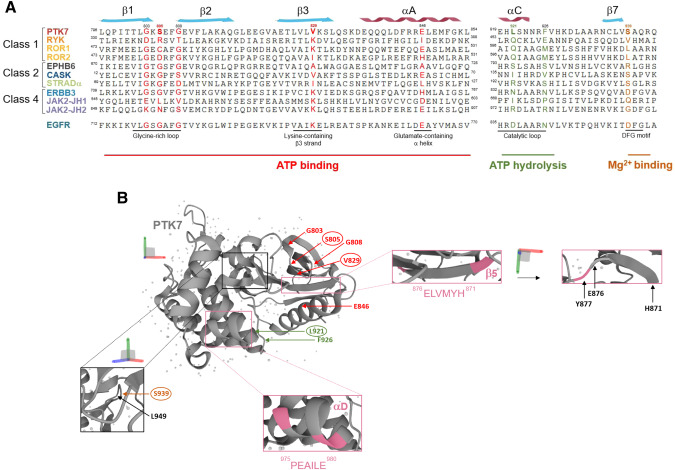
Sequences and organization of pseudokinase domains. **A** Multiple Sequence Alignment (MSA) of key residues situated within the kinase domain of nine pseudokinases in comparison to active EGFR. MSA was performed with the bioinformatics tool ESPript. **B** The 3D structure of the pseudokinase domain of PTK7 was obtained from PDB (ID: 6VG3, residues 774-1069) [7]. The essential residues for ATP binding and hydrolysis, as well as Mg^2+^ binding, are highlighted. The non-conserved residues in PTK7 compared to EGFR are surrounded. The occlusion of the ATP-binding site in PTK7 occurs as a result of the tyrosine Y877 side chain protrusion within the β5/αD hinge and the leucine L949 side chain within the ALG motif.

The concept of pseudokinases emerged 20 years ago when it was realized that about 10% of protein kinases across species have unusual amino acid residues in one or more of the 10 highly conserved and functionally important kinase domain motifs [1, 5]. In particular, four intact motifs comprising 40 conserved residues are necessary in active protein kinase domains: (i) the glycine-rich GXGXXG motif, which allows anchoring of non-transferable phosphates from ATP; (ii) the lysine-containing VAIK motif, in which the lysine residue interacts with the α and β phosphates of ATP to anchor and orient it; iii) the glutamate-residue for a-helix positioning; (iv) the HRD motif, in which the aspartic acid is the catalytic residue; (v) the DFG motif, in which the aspartic acid binds Mg^2+^ that coordinates the β and γ phosphates of ATP in the ATP-binding cleft (Fig. 2A). As detailed in previous reviews, four classes of pseudokinases are defined according to non-canonical consensus motives, alone or in combination (Fig. 1B) [3, 6]. Pseudokinases of Class 1 comprise members characterized by their status as non-ATP and non-Mg^2+^ binders, among those the RTK family members PTK7, RYK, ROR1, and ROR2 (Fig. 1B). However, ROR1 was recently found to bind ATP and be sensitive to chemical inhibitors [7]. The RTK EPHB6 and the cytoplasmic CASK and STRADα are among the protein kinases categorized in the Class 2 pseudokinases in exhibiting ATP binding, without Mg^2+^ coordination due to the replacement of the aspartic residue in the DFG motif within their kinase domains (Fig. 1B). Mukherjee et al. however demonstrated that CASK functions as an active protein kinase even without Mg^2+^ [8]. Mg^2+^-independent ATP binding has also been reported for the pseudokinase Tribble 2 (TRIB2) (not shown) [9]. In an active-like state, the pseudokinase domain of TRIB family members (TRIB1-3) serves as a scaffolding region for ubiquitylation of transcription factors while its C-terminal region is associated with E3 ubiquitin ligases and MAPKK/MEK family members [10]. Class 3 is in the most limited category (not shown) comprising only two members (PEAKs and the *Toxoplasma gondii* protein ROP2) able to bind Mg^2+^ but not ATP [11]. The pseudopodium-enriched atypical kinase (PEAK) family comprises PEAK1 (SgK269), PEAK2 (SgK223) and the recently identified PEAK3. They are categorized as pseudokinases due to alteration of the DGF motif in PEAK1 and PEAK2, and of the HRD motif in PEAK1 and PEAK3. Crystal structures of all PEAKs have unveiled homo- and heterodimerization involving essential N-terminal and C-terminal helices that enclose the pseudokinase domain [12–14]. This dimerization interface, referred as the SHED domain, plays a critical role in the binding of PEAK interacting partners. Specifically, the scaffold protein 14-3-3 forms a heterodimeric complex with PEAK3 in order to regulate its intracellular localization as well as its associated protein network [15]. Collectively, PEAKs serve as molecular scaffolds for assembling signaling complexes that regulate cytoskeleton organization, cell migration and invasion. Finally, the already mentioned ERBB3 and JAK2 are Class 4 pseudokinases that retain the ability to bind ATP and Mg^2+^. However, they have unconventional residues (substitution of the aspartic residue to serine) in their catalytic loop (HRD motif) leading to a much lower intrinsic activity than active kinases, augmented through intramolecular or heteromeric interactions (Fig. 2A) [2, 16].

Of the fifty of so pseudokinases in the human kinome, we focus in this review on the cell surface receptor PTK7, an evolutionary conserved tyrosine Class 1 pseudokinase receptor involved in diverse cancer-related signaling pathways, notably the WNT pathway [17, 18]. According to the phylogenetic tree of protein kinases (Fig. 1A), PTK7 stands alone on a branch that has common ancestors with RTKs of the ALK family, Insulin receptor family, Class 1 pseudokinases ROR1/2, DDR family and RTK family. Less studied and understood than other RTK family pseudokinases such as ERBB3, PTK7 is a very good example of a druggable pseudokinase with strong clinical potential. Indeed, PTK7-dependent signaling has been tightly associated with many aspects of cancer progression and identified as both a marker of poor prognosis and challenged as a promising therapeutic target in several types of cancers [18]. Translational and clinical studies support the implication of PTK7 in tumor development and metastatic dissemination and its suitability as a new therapeutic target using antibody-based strategies, antibody-drug conjugates (ADCs), and chimeric antigen receptor-modified T cells (CAR-T cells).

We report the most recent insights into the activity of PTK7, notably based on x-ray crystal structures of its pseudokinase domain, and the cancer-related pathways associated with the receptor. We also summarize convincing translational studies linking PTK7 to cancer progression and resistance to treatment, and discuss controversial results regarding its role as a poor prognostic marker. Finally, we provide an overview of promising strategies targeting PTK7 as therapeutic interventions suitable also for other pseudokinases.

## From gene discovery to the three-dimensional structure of PTK7

**Fig. 3:**
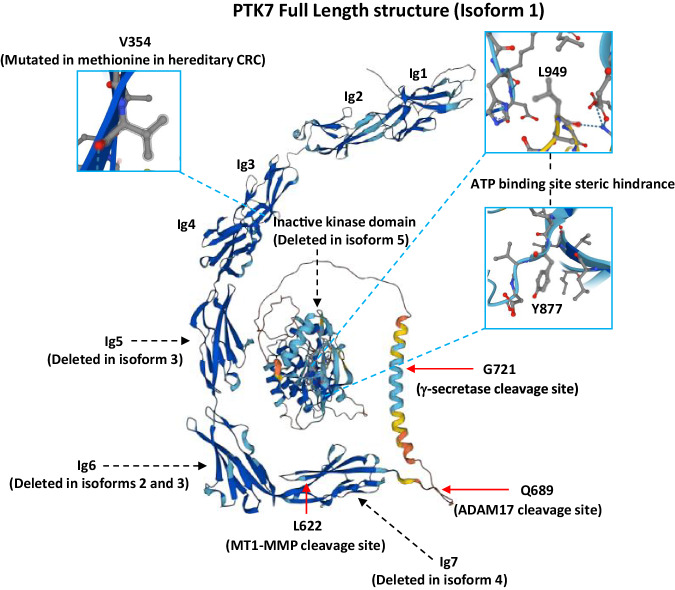
Structure, isoforms, and post-translational modifications of PTK7. The 3D structure of PTK7 based on an AlphaFold model. The picture shows the extracellular mutation found in CRC (Valine 354) and intracellular cleavage sites (Leucine 622, Glutamine 689, Glycine 721) of PTK7 as well as the immunoglobulin domains deleted by alternative splicing, leading to the theoretical generation of five PTK7 isoforms. The picture also details the receptor inactive ATP-binding pocket based on the structure of Lemmon’s lab [7].

The human *PTK7* gene located on chromosome 6p was first cloned from normal human melanocytes in 1993 [19]. Later, Mossie et al. identified PTK7 as a catalytically inactive member of the RTK family upregulated in colon carcinoma and therefore named by the authors Colon Carcinoma Kinase-4 (CCK-4) [17]. Regarding the PTK7 pseudokinase domain, 7 of the 40 consensus residues are unconventional including substitution of the second glycine residue in the G_803_XGXXG_808_ motif with serine or aspartic residues within the catalytic loop and the DFG motif changed to leucine and serine, respectively (Fig. 2A) [19]. In 2020, the crystal structure of the PTK7 kinase domain was solved by Mark Lemmon’s team providing additional and valuable insights into its function (Fig. 2B) [7]. Protein kinases contain an activation loop whose location relative to the ATP-binding site is required for receptor activation through phosphorylation. The PTK7 activation loop conformation closely resembles that of the insulin receptor kinase in its inactive and auto-inhibited form. In addition, the ATP-binding site of PTK7 kinase domain is occluded by the leucine side chains in the ALG motif (L949) and by the projection of a tyrosine side chain from the β5/αD hinge region (Y877) into the adenine ring binding site (Fig. 2B, Fig. 3). However, PTK7 has an active-like “in” αC disposition, which allows the αC/β3 salt bridge to be formed, a hallmark of active kinases [7]. In total, the PTK7 kinase domain has no capacity to bind ATP nor Mg^2+^ and represents a true kinase-dead RTK-like ROR2 and RYK, two other WNT pseudokinase of the Class 1 family [7].

Despite its lack of enzymatic activity, the high conservation of the PTK7 kinase domain sequence across the evolution of species (human, mouse, chicken, *Drosophila, Hydra* among others) suggests an important role in biology. PTK7 is composed of seven extracellular immunoglobulin-like (Ig-like) loops, a transmembrane region and an intracellular kinase domain, and is subjected to alternative splicing events, leading to the expression of different isoforms (Fig. 3). To date, a total of five spliced PTK7 mRNAs have been identified in testicular, colon, liver, and kidney cancer cell lines [20], three of them having either deletions of the fifth, sixth, or seventh Ig-like loops, with a fourth form having a deleted kinase domain (Fig. 3). Recently, a *PTK7* germline variant bearing a missense mutation substituting valine 354 into methionine (V354M) within the fourth Ig-like loop was identified in familial colorectal cancer (CRC) cases and found associated to the oncogenic function of PTK7 by increasing cell proliferation, migration, and invasiveness (Fig. 3) [21].

**Fig. 4:**
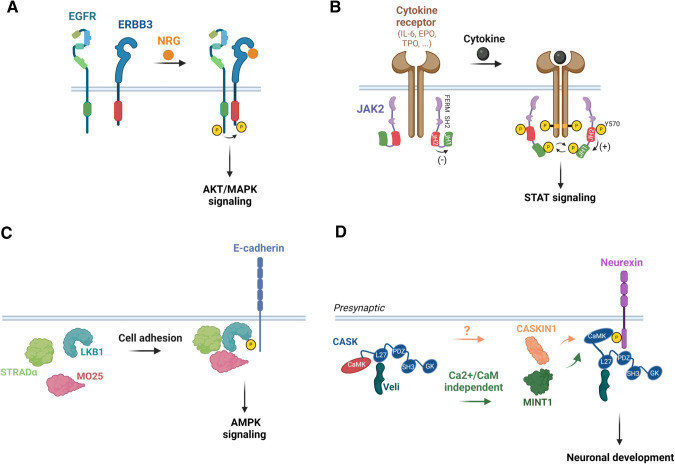
Modes of action of pseudokinases. **A** Neuregulin (NRG) drives EGFR kinase activation through heterodimerization with the pseudokinase receptor ERBB3. **B** JAK2 undergoes conformational changes upon cytokine binding to a specific receptor (IL-6: Interleukine-6, EPO: Erythropoietin, TPO: Thrombopoietin). Receptor-associated JAK2 is activated through autophosphorylation of the tyrosine residue Y570 within its JH2 pseudokinase domain that activates in *cis* the adjacent JH1 kinase domain. This activated-JAK2 complex then phosphorylates tyrosine residues on the receptor, thus generating docking sites for downstream effectors. **C** The pseudokinase STRADα and the scaffolding protein MO25 regulate the activity of the LKB1 tumor suppressor, which in turn governs the function of AMP-activated protein kinase (AMPK). **D** The pseudokinase CASK and Veli form alternative tripartite complexes, associating with either CASKIN1 or MINT1. These complexes potentially facilitate the coupling of CASK to distinct downstream effectors. The pseudokinase domains are depicted in red, while the active kinase domains are colored in green. The figure was created with BioRender.com.

Pseudokinases play a pivotal role in cell signaling by acting as scaffold molecules through heterodimerization with homologous or heterologous molecules, through intramolecular interactions that activate neighboring active kinase domains via allosteric regulation or by assembling protein complexes in cellular functional units (Fig. 4) [5, 22]. As an example of heterodimerization, the pseudokinase ERBB3 interacts at the plasma membrane with active EGFR-related family members in the presence of cognate ligands to amplify the signaling activity of its bound heterologous receptors (Fig. 4A) [2]. Amongst the examples of allosteric regulation, the cytoplasmic JAK2 tyrosine kinase protein contains a pseudokinase domain (JH2) that regulates in *cis* an adjacent active kinase domain (JH1) through conformation changes (Fig. 4B) [16]. A more complex situation is found in the case of the serine/threonine kinase tumor suppressor LKB1 activated in the context of the formation of a heterotrimeric complex comprising the ‘active’ form of the STRADα pseudokinase and the MO25 scaffold protein (Fig. 4C) [23]. Lastly, the N-terminus of CASK is a calcium/calmodulin-dependent protein pseudokinase (CAMK) that plays a role in neuronal development and synapse vesicle transport through its interaction with MINT1 and, in other tissues, in vesicle transport required for insulin secretion (Fig. 4D) [24].

**Fig. 5:**
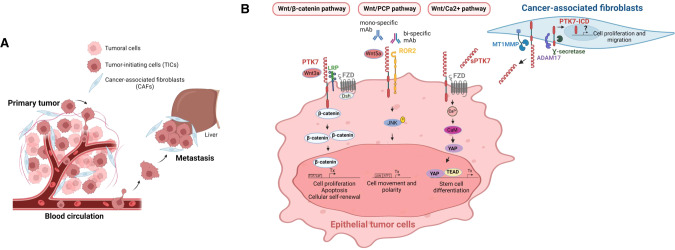
*Cis-* and *trans-*signaling of PTK7. **A** The process of cancer cell dissemination, from tumor initiation to the acquisition of metastatic properties. PTK7 is highly expressed in tumoral cells, and particularly in tumor-initiating cells (TICs). It is predicted to be strongly expressed in metastatic cancer cells. PTK7 is also expressed in endothelial cells forming the blood vessels and in cancer-associated fibroblasts. **B** Enlarged view of cancer cells and CAFs expressing PTK7. Through the interaction with different co-receptors (LRP, FZD, ROR2) and WNT3a/WNT5a ligands, PTK7 signals in *cis* in epithelial cells through three WNT pathways. Mono-/bispecific monoclonal antibodies (mAb) impairing ligand binding or co-receptor function at the cell surface are depicted. Two recent reports demonstrated that soluble PTK7 (sPTK7) released from fibroblasts by the action of ADAM17 and MT1-MMP can trigger WNT/Ca^2+^ *trans-*signaling in epithelial cells (ICD intracellular domain). The figure was created with BioRender.com.

During cancer development, the formation of metastasis, responsible for the greatest number of cancer-related deaths, encompasses tumoral cell detachment from the primary tumor, intravasation into the bloodstream, followed by extravasation and colonization of distant tissues [25–27]. PTK7 is highly expressed in primary tumors and supposedly in metastatic cells but is also found in cancer-associated fibroblasts and endothelial cells. Up to now, most of the studies have depicted the signaling functions of PTK7 in tumoral cells. PTK7 acts as a co-receptor for heterologous active tyrosine kinase receptors and as a scaffolding protein for cytoplasmic signaling molecules. Much like ERBB3, PTK7 is indeed able to form heterodimers with VEGFR and ROR2 RTKs, and participates to signaling upon VEGF and WNT ligand binding, respectively [27–29]. How PTK7 helps these receptors to signal is not clear. Nevertheless, the association of PTK7 with VEGFR, involving probably the extracellular regions of the two partners, increases the affinity of VEGF for its receptor at the surface of endothelial cells, promoting angiogenesis [28]. As in the case of CASK (Fig. 4D), the pseudokinase domain itself may have a docking activity and initiate signaling events through finely tuned protein-protein interactions in the cytoplasm [24, 30, 31]. Likewise, we and others have shown that the PTK7 kinase domain acts as a scaffold domain enabled to interact with cytoplasmic signaling molecules such as β-catenin or RACK1 (Receptor of activated protein kinase C 1), and contributes to WNT signaling (Fig. 5B) [32, 33]. This domain also serves as a docking site for the non-receptor tyrosine kinase Src enabling the downstream effector ROCK2 to modulate actomyosin contractility and WNT/Planar Cell Polarity (PCP) in epithelial cells [34]. Despite having a crystal structure of the PTK7 kinase domain, we do not yet understand the precise molecular mechanism behind these interactions and how these transmit cellular signaling as done with CASK and its partners [24]. Nevertheless, the binding of the PTK7 pseudokinase domain to the armadillo repeats of β-catenin is direct, required for a signaling cascade from the membrane to the nucleus, and amenable to pharmacological inhibition [32, 35].

## Signaling pathways linked to PTK7 in cancer

### Role of PTK7 in the WNT/PCP pathway

PTK7 is as a bona fide regulator of PCP, a developmental process driven by a subset of WNT ligands necessary to coordinate cell behavior across a two-dimensional sheet in tissues, orthogonally to the axis of apico-basal polarity [36]. Mutations in WNT/PCP genes *(PTK7, CELSR*, *Frizzled*, *VANGL*, *Dishevelled*, *Prickle)* contribute to the etiology of neural tube closure defects, including in its most severe form called craniorachischisis in mice, *Xenopus*, and humans [37, 38]. In addition, PTK7 mutant zebrafish develop three-dimensional curvature of the spine at juvenile stages [39].

Beyond the genetic and functional interactions observed between PTK7 and other WNT/PCP genes during embryogenesis, PTK7 modulates WNT/PCP receptors in cancer cells. Indeed, PTK7 associates with ROR2 and the non-canonical WNT ligand WNT5a promoting actin cytoskeleton reorganization and cell movements in a JNK-dependent manner (Fig. 5B) [29]. This heterotrimeric complex is conserved in *Xenopus* where it regulates protocadherin *papc* expression and embryonic development [29]. However, it signals differently in other cellular systems such as pro-B cells [40] or ovarian cancer (OVCA) cells where PTK7 modulates cell adhesion and Rho-GTPase signaling to sustain epithelial-mesenchymal transition and cell plasticity [41].

PTK7 also intersects with the WNT/PCP signaling pathway by recruiting the downstream effector Dsh *(Dishevelled)* to the plasma membrane and by being part of a Fz7 *(Frizzled 7)*/Dsh complex regulating neural crest migration in *Xenopus* embryos [42]. This function is mediated through interaction of the pseudokinase domain of PTK7 with RACK1, an intracellular adapter protein interacting with PKCδ1 *(Protein kinase C delta 1)* that promotes the recruitment of Dsh to the plasma membrane [33].

### Role of PTK7 in the WNT/β-catenin pathway

In contrast to the well-established implication of PTK7 in the WNT/PCP pathway, its function in the WNT/β-catenin pathway is more controversial. This signaling pathway is involved in the early stages of cancer as alterations of components such as APC *(Adenomatous polyposis coli)*, β-catenin, or Axin are found in 93% cases of CRC [43]. Most, if not all, of these mutations induce constitutive activation of the WNT/β-catenin signaling by cytosolic accumulation of β-catenin and translocation into the nucleus where it interacts with transcription factor TCF/Lef *(T-cell factor/lymphoid enhancer factor)* family members leading to chromosomal instability, cell proliferation, and inhibition of cell differentiation. As mentioned earlier, PTK7 directly interacts with β-catenin and promotes downstream events induced by the WNT3a ligand in mammalian cells and *Xenopus* (Fig. 5B) [32]. We recently reconstituted the PTK7-β-catenin interaction using an *in cellulo* NanoBRET (Bioluminescence Resonance Energy Transfer) format and identified small-molecule inhibitors able to disrupt this protein-protein interaction and downregulate WNT signaling similarly to PTK7 knockdown [32, 35]. Our observation in CRC cells was not confirmed in normal cells, probably due to the different landscape of WNT co-receptors and ligands associated to PTK7 [44]. In *Xenopus* embryos, PTK7 indeed interacts with LRP6 via its transmembrane domain and, like a molecular switch, promotes at the same time WNT/β-catenin signaling induction and WNT/PCP signaling repression [45]. Interestingly, the canonical WNT/β-catenin pathway, which plays a pivotal role during tumor initiation and the early stages of tumor progression by regulating cancer cell proliferative and stemness properties, can be turned down by upregulation of WNT/PCP signaling in more advanced stages where cell invasiveness and resistance to treatments are observed [18].

### Role of PTK7 in WNT-independent signaling pathways

The role of PTK7 is not restricted to WNT signaling. Indeed, PTK7 genetically interacts with Plexins, acting in a pathway important for *Drosophila* axon guidance and *Xenopus* neural crest cell migration [46]. PTK7 expression is elevated in human vascular endothelial cells in which PTK7 interacts with the RTK FLT-1/VEGFR1 and promotes its phosphorylation, AKT activity and VEGF-induced cell migration and angiogenesis [28, 47]. PTK7 also activates angiogenesis by modulating KDR/VEGFR2, particularly its oligomerization [27]. Notably, anti-PTK7 monoclonal antibodies (mAbs) inhibit VEGF-induced angiogenesis by blocking PTK7-KDR interaction [48]. While only used in vitro, mAbs targeting the extracellular domain of PTK7 hold promise in impeding its dimerization or heterodimerization with co-receptors like ROR2 or ligands such as WNT3A and WNT5A (Fig. 5B). Additionally, bispecific mAbs could be developed in the future to simultaneously inhibit PTK7 and ROR2 signaling.

### Cleavage, nuclear translocation, and shedding of PTK7

PTK7 signaling is regulated by proteolytic cleavage events that involves MT1-MMP (*Membrane Type-1 Matrix MetalloProteinase)*, a well characterized enzyme highly expressed during cancer cell invasion and playing a central role in extracellular matrix degradation [49]. MT1-MMP cleaves PTK7 at residue leucine 622 (L622) located within the seventh Ig-like loop (Fig. 3) [50], promoting the release of soluble extracellular PTK7 (sPTK7) and preparing the remaining membrane-bound PTK7 for two further cleavages: one performed by the disintegrin and metalloprotease ADAM17 at residue glutamine 689 (Q689) close to the transmembrane region, and one by the γ-secretase at residue glycine 721 (G721), leading to the cytosolic release of the PTK7 intracellular domain (Fig. 3) [51]. In CRC and fibrosarcoma cells, the cleaved intracellular domain translocates into the nucleus to promote cell migration and proliferation [51, 52]. Recently, Yun et al. discovered that sPTK7 released by senescent fibroblasts induces intestinal stem cell differentiation in a paracrine manner through the WNT/Ca^2+^ pathway (Fig. 5B) [26]. Similarly, *Drosophila* sPTK7 released by enteroendocrine cells was shown to dimerize with full-length PTK7 present at the plasma membrane of intestine stem cells, promoting their directed migration to sites of injury through the non-canonical WNT signaling [53]. Thus, cleaved forms of PTK7 appear to have cell autonomous and non-cell autonomous biological properties.

## Translational studies linking PTK7 to cancer progression and resistance to treatment

Numerous studies have demonstrated overexpression of PTK7 in solid and hematological tumors and strong correlation with poor prognosis and resistance to treatment, suggesting that this receptor may represent a valuable biomarker and/or therapeutic target [18]. However, thus far, the studies have reported its upregulation in numerous primary tumors but have not comprehensively studied its deregulation along the whole process of cancer development, spanning from primary tumor formation to metastasis dissemination (Fig. 5A). High expression of PTK7 in lymph nodes is associated with poorer disease-free survival in primary breast cancer (BC) [54] and in triple-negative breast cancer (TNBC) patients whose tumors lack expression of estrogen and progesterone receptors and overexpression of HER2 [55]. Similarly, overexpression of PTK7 is linked to cancer dissemination in non-small cell lung cancer (NSCLC) [56], OVCA [57], oral tongue squamous cell carcinoma (OTSCC) [58], thyroid cancer [59], acute myeloid leukemia (AML) [60], liposarcoma [61], esophageal squamous carcinoma [62], hepatic [63], and cervical cancers [64]. On the contrary, PTK7 is a favorable prognostic marker in stages II and III gastric cancers [65].

Notwithstanding, most of the functional studies conclude that PTK7 overexpression promotes cell proliferation and induction of pro-migratory and pro-metastatic phenotypes in vitro and/or in vivo [25, 52, 59–62, 66–68]. PTK7 may also induce resistance to anthracycline-based chemotherapy in AML and BC cells. The potential of PTK7 as a therapeutic target has been demonstrated in AML and in TNBC cells in which recombinant sPTK7 and anti-PTK7 antibodies, respectively, reverse PTK7 pro-tumorigenic effects [54, 55, 60].

However, low expression of PTK7 linked to a positive or negative clinical outcome has also been described in cancer [69], making the situation more complex than expected. Expression of PTK7 is decreased or lost in metastatic melanoma, possibly correlating with deletion of chromosome 6p where the gene is located [70], indicating a putative action of PTK7 as tumor suppressor under certain circumstances [71]. PTK7 expression is also decreased in ovarian epithelial neoplasms with poor prognosis [72], in invasive ductal BC cells [69], and in human lung squamous cell carcinoma (LSCC) [73]. In non-metastatic patients with CRC, upregulation of PTK7 correlated with reduced metastasis-free survival [25], whereas Tian et al. reported favorable overall survival [74]. This discrepancy is probably linked to particular tumoral molecular contexts and interacting partners, and/or to the diverse forms of PTK7 generated by post-transcriptional/translational regulations [20, 21, 52]. However, none of these possibilities have been explored yet. Interestingly, Golubkov et al. reported that the ratio of full-length PTK7 to cleaved PTK7, rather than full-length PTK7 alone, was a useful biomarker in malignancies, providing a possible interpretation of the contradictory conclusions drawn by merely assessing full-length PTK7 expression alone [52]. Whereas in some cancer contexts the role of PTK7 has been corroborated by functional studies, in others, correlations of PTK7 levels with prognosis would benefit of functional assays to unequivocally determine whether PTK7 acts as a positive or negative regulator of tumorigenicity.

Lastly, in NSCLC, membrane-localized PTK7 interacts with and stabilizes NDRG1 to promote EGFR-tyrosine kinase inhibitor resistance [75]. Moreover, in melanoma, BET inhibitors disrupt the cell surface interaction between PTK7 and AMIGO2 by promoting the proteolytic processing of PTK7 [76]. These studies highlight novel aspects of PTK7 regulation and involvement in response and resistance to cancer treatments as found for other tyrosine kinase receptors such as DDR1 [77].

## Targeting PTK7 in cancer

Targeting pseudokinases remains a challenge in therapeutic development [2]. The lack of enzymatic activity and its context-dependent action at the plasma membrane and in the nucleus have limited the development of agents counteracting PTK7 functions. Most of the established strategies are designed in relation to its enhanced expression in tumors, employing antibody-based drug conjugates.

### Drug conjugates targeting PTK7-positive tumors

**Fig. 6:**
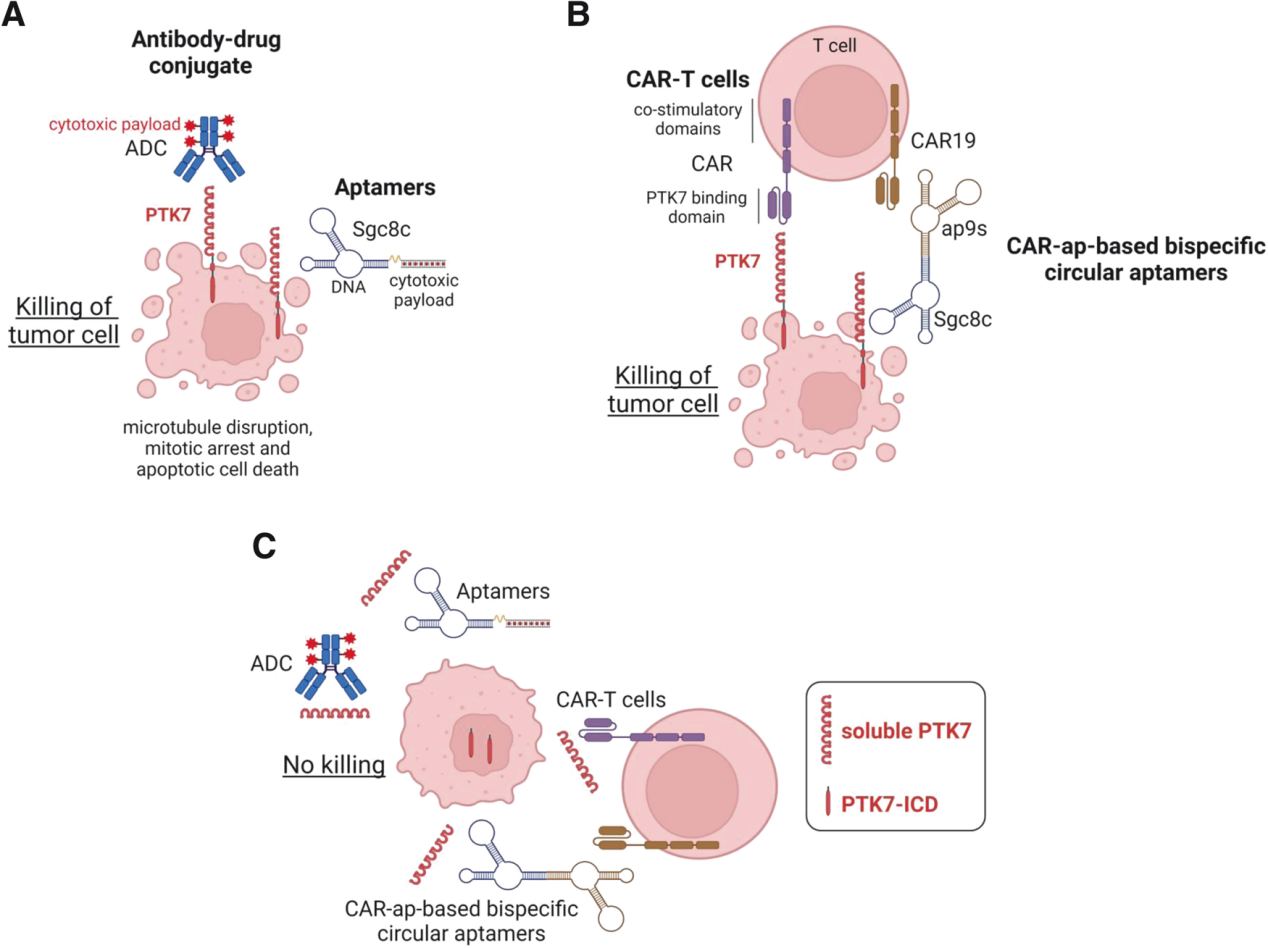
Therapeutic strategies killing PTK7-overexpressing tumors. Four approaches can target tumor cells overexpressing PTK7 at their cell surface: **A** ADC (ongoing clinical trials) and aptamer coupled to cytotoxics, **B** CAR-T cells, and CAR-ap-based bispecific circular aptamers. **C** Cleavage of PTK7 leading to the production of soluble PTK7 and nuclear PTK7-ICD leads to resistance of therapeutic strategies targeting surface-bound PTK7. The figure was created with BioRender.com.

A strategy targeting PTK7 that has reached clinical phase consists of using an Antibody-Drug Conjugate (ADC), developed as PF-06647020/cofetuzumab pelidotin (Fig. 6A) [57]. ADCs combine the tumor selectivity of antibodies with the therapeutic potency of linked cytotoxic or cytostatic small molecules to limit the systemic side effects of the chemotherapy. PF-06647020 is an ADC comprising a humanized anti-PTK7 monoclonal antibody (mAb) linked to an auristatin microtubule inhibitor payload by a cleavable valine-citrulline based linker (Table 1) [57, 78–80]. Preclinical studies have shown sustained tumor regression in patient-derived xenograft models of TNBC, NSCLC, and OVCA with greater antitumor activity than standard chemotherapy, and reduced frequency of TICs (tumor-initiating cells) [57]. A first-in-human phase I study of PF-06647020 in patients with advanced solid tumors resistant to, or with no available, standard therapy was thereafter completed. This study demonstrated a tolerable safety profile and preliminary clinical activity of this ADC in patients bearing PTK7-positive solid tumors. Further clinical investigations are ongoing to assess its therapeutic potential [81]. Another phase I study combining PF-06647020 with an inhibitor of PI3K/mTOR signaling was conducted for metastatic TNBC (NCT03243331), however the results are not yet available.

**Table 1:** Compounds targeting Class 1/2 pseudokinases of the RTK family.

Target gene	Compound	Cancer	Trial number	Description	Trial phase	Refs
**PTK7**	ADC	NSCLC, TNBC, OVCA	NCT02222922	Cofetuzimab pelidotin: auristatin linked anti-PTK7 monoclonal antibody	Phase I	[57]
	Aptamer	ALL	–	Doxorubicin coupled to PTK7-targeting Aptamer	–	[83]
	CAR-T	NSCLC, OVCA, Neuroblastoma	–	Chimeric antigen receptor T (CAR-T) cells expressing PTK7-targeting ScFv sequence	–	[86–89]
	Allogenic CAR-T	BC, NSCLC, CRC, pancreatic cancer, OVCA	–	CRISPR/Cas9 genome editing of T cells	–	[92]
	CAR-T-aptamer	ALL	–	CAR-T cells expressing PTK7-targeting aptamer	–	[87]
	Small molecule	CRC	–	Small molecule preventing PTK7/β-Catenin interaction	–	[35]
**ROR1**	Kinase inhibitor	Chronic Myeloid Leukemia (CML), ALL	–	Ponatinib	–	[7]
	mAb	Chronic Lymphocytic Leukemia (CLL)	NCT02222688	Cirmtuzumab	Phase I	[95]
	ADC	Hematological malignancies	NCT03833180	Monomethyl auristatin (MMAE) linked anti-ROR1 monoclonal antibody	Phase I	[96]
	CAR-T	CLL, ALL, NSCLC, TNBC	NCT02706392	CAR-T cells expressing ROR1 targeting ScFv sequence	Phase I	[97]
**ROR2**	ADC	NSCLC, TNBC	NCT03504488	MMAE linked anti-ROR2 monoclonal antibody	Phase I/II	[98]
	CAR-T	Renal Cell Carcinoma	NCT03393936	CAR-T cells expressing ROR2 targeting ScFv sequence	Phase I/II	[98]
**RYK**	mAb	BC	–	Monoclonal antibody preventing Wnt5A-mediated RYK activation	–	[99]
**EPHA10/EPHB6**	Kinase inhibitor	–	–	Type I (Dasatinib) and Type II (AMG Tie2-1)	–	[100]

An alternative strategy in development uses a similar approach with aptamers, also known as “chemical antibodies”, instead of anti-PTK7 mAbs (Fig. 6A). Aptamers are small single-strand (ss) RNA or DNA sequences with particular secondary and tertiary structures capable of binding to a specific target. They are selected by the SELEX (Systematic Evolution of Ligands by Exponential Enrichment) from random libraries of ssDNA or ssRNA molecules [82] and present several advantages compared to mAbs, such as limited immunogenicity thus fewer allergic reactions, low molecular weight improving tumor penetration, and are easily modified by chemical reactions. Aptamers have emerged as innovative tools to recognize cancer-related antigens in targeted therapy, with promising potential for diagnostic and therapeutic uses as delivery systems for drugs. Aptamers against the extracellular region of PTK7 have been identified and one of them, an ssDNA sequence composed of 42 nucleotides named Sgc8c, was demonstrated to specifically eradicate Acute Lymphoblastic Leukemia (ALL) cells when coupled to doxorubicin, a drug used to treat this disease (Table 1). In vitro studies have shown the potential of this targeted treatment against ALLs or other PTK7-positive tumors, although no evidence of in vivo efficacy has been demonstrated yet [83, 84].

### Immunotherapy

Adoptive cell transfer immunotherapies such as chimeric antigen receptor T (CAR-T) or NK (CAR-NK) cell therapies are amongst the most promising treatments in oncology [85]. Recently, eradication of PTK7-overexpressing tumors by CAR-T cell therapy has been proposed in several studies (Fig. 6B) [86–89]. CAR-T cells are genetically modified T lymphocytes forced to express a chimeric receptor (CAR), typically a single-chain variable fragment of a mAb, to confer T cells with “de novo” defined specificity against a particular tumoral antigen found in the patient, fused to a hinge region, transmembrane and intracellular signal transduction domains [90]. Previous studies have demonstrated that this strategy can induce durable and complete responses in patients with hematological malignancies, although its efficacy in solid tumors remains limited [91]. PTK7-targeting CAR-T cells exhibited in vitro antigen-specific cytokine production and cytotoxicity against multiple PTK7-positive cell lines derived from solid tumors. In vivo, PTK7-targeting CAR-T cells induced significant inhibition of tumor growth and prolonged overall survival in xenograft tumor models of lung cancers. Further studies will have to assess its antitumor efficacy in clinically relevant settings [86]. Additionally, allogenic anti-PTK7 CAR-T cells have been generated using CRISPR/Cas9 genome editing of T cells from healthy donors. These anti-PTK7 CAR-T cells showed efficacy in vitro and in various solid tumor engrafted in mice (Table 1) [92]. One of the most recent developments was the design of CAR-specific binding aptamers (CAR-ap) (Fig. 6B) that successfully combine for the first time the specificity of aptamers with the potency of immune effectors to selectively target PTK7 in the context of hematological malignancies (Table 1) [87]. The bispecific CARs, based on circular aptamers, represent cost-and therapeutically effective tools. They provide the advantage of guiding CAR-T cells towards tumor cells, thereby enhancing their antitumor cytosolic activity through the bridging mechanism between CAR-T cells and tumor cells.

Of note, as the extracellular domain of PTK7 is cleaved by the MT1-MMP and ADAM17 metalloproteinases [50, 51], we foresee that tumoral cells with high cleavage activity could become resistant or escape ADC/CAR-T/ap therapies and that measurement of sPTK7 in the serum of patients would have added value in the monitoring of treated patients (Fig. 6C).

### Small molecules targeting PTK7

**Fig. 7:**
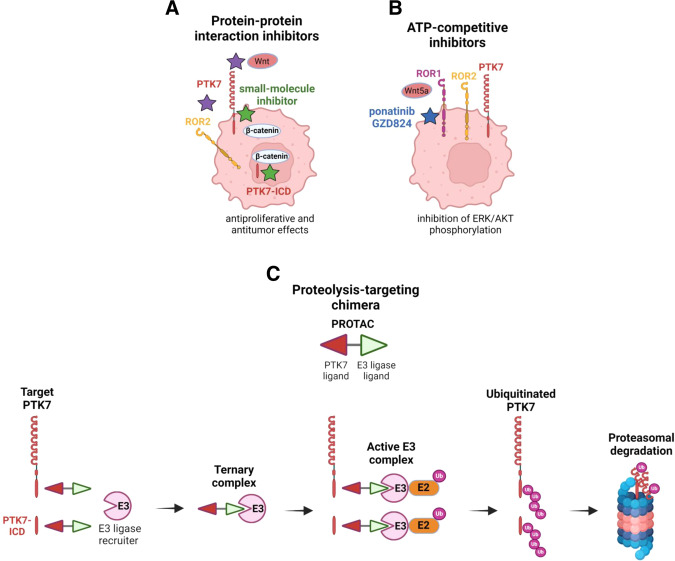
Design of modulators of PTK7 function or expression. Possible approaches to weaken PTK7 signaling using either **A** small-molecule inhibitors interfering with its binding partners. Green stars represent PTK7/β-catenin inhibitors, while the purple ones represent potential protein-protein interaction inhibitors, **B** ATP-competitive inhibitors specific for ROR1, and **C** compounds leading to PTK7 proteasomal degradation by the proteolysis targeting chimera (PROTAC) method. Covalently-linked PTK7 and E3 ligase ligands engage interaction with their respective partners in cells. Recruitment of the E2-Ub complex by the E3 ligase results in ubiquitinylation of PTK7 and subsequent degradation by the proteasome. The figure was created with BioRender.com.

We and others have accumulated evidence that the PTK7 kinase domain is endowed with signaling functions linked to cancer despite its deficient catalytic activity, probably through signalling docking property [32–34, 51, 93]. We identified β-catenin as a direct partner of PTK7 and demonstrated the requirement of this interaction in WNT/β-catenin signaling regardless of the mutational status of *APC* and *CTNN1* genes [32, 35]. Through a multi-screening strategy based on a newly developed NanoBRET assay recapitulating the interaction between PTK7 and β-catenin that combined virtual screening, high throughput screening, and repurposing of drugs in development, we demonstrated that inhibition of this protein-protein interaction is achievable with small molecules. The selected compounds had inhibitory activity at micromolar range potency, although with slightly different mechanisms when considering the expression of WNT target genes in the CRC cells tested [35]. Remarkably, in vitro, the PTK7/β-catenin inhibitors impaired proliferation and anchorage-independent cell growth. The protein interaction surfaces between PTK7 and β-catenin thus offer opportunities for new therapeutic strategies to inhibit CRC cell growth dependent on WNT signaling pathway (Fig. 7A) [35]. Further potency optimization is necessary for these compounds prior to biological activity testing using patient-derived models in ex vivo and in vivo settings. Furthermore, as PTK7 is prone to cleavage and nuclear translocation, these inhibitors offer the added benefit of targeting the PTK7/β-catenin interaction within the nucleus (Fig. 7A). Other strategies would consist in identifying inhibitors that can bind to the extracellular domain of PTK7, thereby suppressing PTK7 interaction with WNT ligands or cell surface co-receptors (Fig. 7A). The determination of the x-ray crystal structure of PTK7 showed an unusual conformation of its pseudokinase domain described as “pseudo-active” that offers other potential pharmacological options to explore for future drug development such as protein-protein interaction inhibitors or PROTAC molecules (Fig. 7B, C) [7]. Small molecules that target the ATP-binding site of pseudokinases have been documented [2]. These inhibitors can modulate inter- and intramolecular interactions as observed in the case of ponatinib or GZD824 for ROR1, which will be discussed thereafter (Fig. 7B) [7]. Identification of chemical PTK7 ligands able to bind its kinase domain could also prove beneficial for proteasomally mediated degradation of PTK7 via PROTAC, as reported for ERBB3 (Fig. 7C) [94]. Resolution of the three-dimensional structure of the PTK7 pseudokinase domain with its cognate partners (β-catenin, RACK1,..) would also greatly help in the design of specific inhibitors able to counteract PTK7 signaling in cancer cells.

## Conclusion

As more interest develops in the impact of pseudokinases in cancer, deeper knowledge of their structural and functional properties remains crucial for developing specific and clinically valuable therapies. A key step for the selection of novel drugs relies on a better understanding about their mode of action in the context of their interaction with binding partners or, when possible, in the presence of oncogenic mutations, as done with Class 4 pseudokinases JAK2 and ERBB3 [2]. In the case of less-known Class 1 and 2 pseudokinases, more work is needed to achieve such therapeutic successes in hematological and solid tumors.

The discovery of the structural and dynamic properties of the Class 1 pseudokinase domains of WNT receptors (ROR1, ROR2, RYK, PTK7) by Sheetz et al., added to drug screening, has allowed to identify ROR1 as a target of the small-molecule ponatinib, a kinase inhibitor approved by the Food Drug Administration in chronic myeloid and acute lymphoblastic leukemia, opening up wider perspectives of clinical development (Fig. 7B) [7]. Other strategies targeting ROR1 are currently ongoing with antibody-based therapies (Table 1) [95]. Notably, cirmtuzumab, an anti-ROR1 mAb, reached phase I clinical trial in patients with relapsed chronic lymphocytic leukemia (LCC) [95]. Clinical trials also evaluated the ADC VLS-101 and anti-ROR1 CAR-T cells for the treatment of hematological cancers [96, 97]. Despite strong evidence about the oncogenic role of ROR2, therapeutic interventions against this RTK are still at their early stages. Nevertheless, anti-ROR2 ADCs and ROR2-specific CAR-T cells are clinically evaluated in solid malignancies and in phase I/II in renal cell carcinoma, respectively [98]. RYK-targeted therapies have been so far poorly explored and are limited to the development of mAbs able to inhibit signaling in response to WNT5a, a RYK ligand [99]. Such strategies could be developed against PTK7 in the future, providing that its interaction with its ligands is better understood (Fig. 5B). Concerning the poorly characterized Class 2 pseudokinase receptors EPHA10 and EPHB6 whose expression is often disregulated in cancer, their ability to bind kinase inhibitors offer opportunities for future therapeutics [100].

Since its discovery, PTK7 has drawn the interest of many labs for its implication in diseases, including cancer [18]. Early work by our team elucidated the prominent role of PTK7 in drug resistance in leukemia [60] and later on in colon cancer [25]. Over the past 10 years, its importance has been extended to other malignancies. As a membrane receptor overexpressed in aggressive cancers, PTK7 is currently targeted by ADC and CAR-T cell-based therapies [57, 78, 86]. However, these promising avenues merit further exploration, keeping in mind that the expression of PTK7 is not limited to tumor cells and that a better understanding of its role in non-tumoral cell populations is needed to anticipate possible adverse effects during the treatment. Furthermore, abundant expression of PTK7 has been detected in the vast majority of primary cancer samples; yet a comprehensive exploration of its deregulation along the entire tumorigenesis process is missing (Fig. 5A). Further investigation should address the expression pattern of PTK7 and its signaling properties at all steps of cancer dissemination.
